# Social defeat stress causes depression-like behavior with metabolite changes in the prefrontal cortex of rats

**DOI:** 10.1371/journal.pone.0176725

**Published:** 2017-04-28

**Authors:** Yi-Yun Liu, Xin-Yu Zhou, Li-Ning Yang, Hai-Yang Wang, Yu-Qing Zhang, Jun-Cai Pu, Lan-Xiang Liu, Si-Wen Gui, Li Zeng, Jian-Jun Chen, Chan-Juan Zhou, Peng Xie

**Affiliations:** 1 Department of Neurology, The First Affiliated Hospital of Chongqing Medical University, Chongqing, China; 2 Chongqing Key Laboratory of Neurobiology, Chongqing Medical University, Chongqing, China; 3 Institute of Neuroscience and the Collaborative Innovation Center for Brain Science, Chongqing Medical University, Chongqing, China; 4 School of Public Health and Management, Chongqing Medical University, Chongqing, China; Radboud University Medical Centre, NETHERLANDS

## Abstract

Major depressive disorder is a serious mental disorder with high morbidity and mortality. The role of social stress in the development of depression remains unclear. Here, we used the social defeat stress paradigm to induce depression-like behavior in rats, then evaluated the behavior of the rats and measured metabolic changes in the prefrontal cortex using gas chromatography-mass spectrometry. Within the first week after the social defeat procedure, the sucrose preference test (SPT), open field test (OFT), elevated plus maze (EPM) and forced swim test (FST) were conducted to examine the depressive-like and anxiety-like behaviors. For our metabolite analysis, multivariate statistics were applied to observe the distribution of all samples and to differentiate the socially defeated group from the control group. Ingenuity pathway analysis was used to find the potential relationships among the differential metabolites. In the OFT and EPM, there were no significant differences between the two experimental groups. In the SPT and FST, socially defeated rats showed less sucrose intake and longer immobility time compared with control rats. Metabolic profiling identified 25 significant variables with good predictability. Ingenuity pathways analysis revealed that “Hereditary Disorder, Neurological Disease, Lipid Metabolism” was the most significantly altered network. Stress-induced alterations of low molecular weight metabolites were observed in the prefrontal cortex of rats. Particularly, lipid metabolism, amino acid metabolism, and energy metabolism were significantly perturbed. The results of this study suggest that repeated social defeat can lead to metabolic changes and depression-like behavior in rats.

## Introduction

Major depressive disorder (MDD) is a serious mental disorder with high morbidity and mortality, and is a worldwide public health concern [1–3]. In recent years, the number of patients with depression has increased dramatically, and it may become the second leading cause of disability worldwide by 2020 [4]. The primary focus of the current research is to investigate the correlation between the occurrence of stressful life events and the onset of MDD [5, 6]. The relationship between them is probably causal, but not all people who encounter a stressful life experience show depressive behaviors [7, 8]. However, it remains unclear to what extent stressful life events cause depressive behaviors and what molecular alterations they may cause in the brain.

Various animal models have been used to study the molecular mechanisms of MDD [9–12]. Among these, the social defeat procedure appears to be one of the best methods to evaluate depressive behavior, and is based on a robust theoretical paradigm [10, 13]. Conventional animal models of MDD, which include procedures such as immobility, cage tilt, stroboscopic illumination, and inescapable electric footshock, do not adequately mimic human experience. In real-life situations, people frequently encounter stimuli generated from the interaction with other people, and social challenge appears to be the most prevalent stressor in humans and social animals [14–17].

Recent studies have demonstrated that mental and behavioral changes in patients with MDD are associated with significant metabolic changes [18–21]. Metabolites play an important role in biological systems, and are the building blocks of numerous biological components, such as proteins, RNA, and DNA. Moreover, metabolites play critical roles in regulation and signaling. Metabolomics, a powerful analytical method in systems biology, allows researchers to study global changes in low-molecular-weight metabolites. It has been extensively used to characterize metabolic changes in various diseases, and it facilitates the identification of disease signatures that can serve as biomarkers [22, 23]. Compared with other “-omic” strategies, the core advantages of metabolomics are the close biological proximity to the phenotype of the system and the ability to observe rapid perturbations in the metabolome [24]. In previous studies, we found a significant metabolite disturbance in plasma and urine of patients with MDD, schizophrenia or bipolar disorder [20, 25–27]. Moreover, postmortem and neuroimaging studies have reported that gray matter volume and glial density in the prefrontal cortex are significantly reduced in MDD patients [28, 29]. The prefrontal cortex is thought to mediate the cognitive aspects of depression, such as feelings of worthlessness and guilt [19]. However, published studies have been unable to clarify the molecular alterations after social stress, and only a few studies have undertaken comprehensive metabolic profiling in the brain.

In this study, we aim to study the effects of social defeat stress on behavior and brain metabolites. First, we used the social defeat stress paradigm to induce depression-like behavior, and identified metabolic changes in the prefrontal cortex using gas chromatography-mass spectrometry (GC-MS). Second, we used multivariate statistics and ingenuity pathway analysis (IPA) in data processing to find potential biological functions among the differential metabolites.

## Materials and methods

### Animals and ethical statement

Male Sprague–Dawley (SD) rats, with initial weights of 250–300 g, were obtained from the animal facility at Chongqing Medical University (Chongqing, China), and served as experimental intruder or control animals. They were housed in individual cages for 10 days before the beginning of the experiments. Male Long-Evans (LE) rats, weighing 380–450 g, were obtained from the animal facility at Third Military Medical University of Chinese People’s Liberation Army (Chongqing, China), and served as resident rats. These male LE rats were housed in cages (length: 54 cm; width: 40 cm; height: 20 cm) together with an oviduct-ligated female rat for 10 days. Throughout the experiment, all rats were maintained under standard laboratory conditions (21 ± 1°C, 55 ± 5% relative humidity, and a 12–12h light–dark cycle with lights on at 19:00) with food and water available ad libitum. All animal handling and procedures followed the recommendations of the Guide for the Care and Use of Laboratory Animals, and were approved by the Ethics Committee of Chongqing Medical University.

### Experimental procedures

#### Social defeat procedure

**Fig 1:**
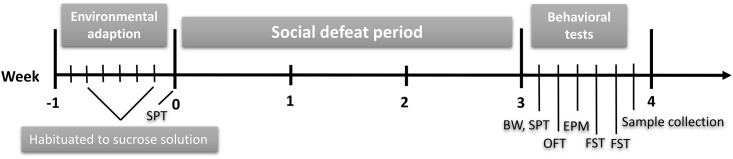
Experimental flowchart. BW, body weight; SPT, sucrose preference test; OFT, open field test; EPM, elevated plus-maze; FST, forced swimming test.

The social defeat procedure was adapted from previous studies with minor modifications [30, 31]. All animals were adapted to standard conditions for 1 week before commencement of the social defeat procedure (Fig 1). The social defeat procedure started at 09:00 under a dim red light. Each experimental SD rat was transferred from its home cage and introduced into the resident’s cage. Ten minutes before the process, the female LE rats were removed from the cages. Each procedure consisted of two consecutive periods (total 60 min). During period 1, intruders were placed in the home cage of aggressive LE male rats for a physical confrontation (5 min). When the intruders adopted a freezing behavior or submissive posture for approximately 5 s during this period, the intruders were separated with a wire-mesh protective cage (10 × 10 × 15 cm) that was placed in the resident’s cage. During period 2, the intruders were kept in the wire-mesh protective cage for 55 min. In the protective cages, the intruders were allowed full visual, olfactory, and auditory exposure to the residents without physical interaction. Intruders in the control group were exposed to the empty home cage of aggressive LE rats for 60 min continuously.

Intruders were subjected to the social defeat exposure once daily, five times each on weeks 1 and 3, and once daily, three times, on week 2. Body weights were measured before the social defeat exposure and weekly throughout the social defeat period. Within the first week after the social defeat procedure, the sucrose preference test (SPT), open field test (OFT), elevated plus maze (EPM) and forced swim test (FST) were conducted to examine the depressive-like and anxiety-like behaviors. The behavioral tests took place in a soundproof room between 09:00 and 15:00 under a dim red light.

#### Sucrose preference test

Before conducting the sucrose preference test (SPT), rats were habituated to 1% sucrose (w/v) for 5 days, and the position of the bottle was alternated across animals and days to reduce side bias. On the testing day, rats were deprived of water and food for 6 h and then presented with preweighed identical bottles of 1% sucrose and water. All fluid consumptions were recorded by weighing the two bottles before the test and 1 h after the start of the test. Sucrose preference was calculated as the sucrose preference % = (sucrose intake/total intake) × 100%.

#### Open field test

The open field test (OFT) was conducted with an automated video tracking system (SMART; Panlab SL, Barcelona, Spain) that contained a square polyvinyl carbonate apparatus (100 × 100 × 40 cm). Total distance, time spent in the center, and the number of rearing behaviors (defined as an upright posture maintained on hind paws) were measured during a predetermined period of time (5 min).

#### Elevated plus maze

The elevated plus maze (EPM) apparatus consisted of four elevated arms (50 cm above the floor, 50 cm long, and 10 cm wide), with two open arms and two closed arms with 40-cm-high walls. Activity within the two open and two closed arms of the EPM was measured over a 5-min period. Frequency and time spent in the open and closed arms were measured by two blinded observers.

#### Forced swim test

For the forced swim test (FST), rats were individually placed into glass cylinders (50 cm high, 20 cm in diameter) containing 30 cm of water at 23 ± 2°C for 15 min, and then gently dried and returned to their home cages. They were placed again in the cylinders 24 h later, and the 5-min forced swim test was conducted. All sessions were recorded with an automated video tracking system (SMART; Panlab SL). Immobility was defined as the least amount of movement needed to stay afloat.

### Sample preparation

After the behavioral tests were completed, rats were deeply anesthetized with 10% chloral hydrate (100 g/0.4 ml i.p.), and then sacrificed by dislocation of the cervical vertebrae. The whole brain was quickly removed, and the prefrontal cortex was dissected from the brain, rapidly frozen with liquid nitrogen, and then stored at −80°C until analysis. For GC-MS analysis, a 40-mg brain tissue sample was homogenized after adding 20 μL internal standard solution (L-2-chlorophenylalanine, 0.03 mg/mL) and 500 μL methanol-water-chloroform (5:2:2, v/v/v). The mixture was sonicated for 5 min and incubated for 20 min at 4°C, then centrifuged at 14 000 g for 15 min at 4°C. A 300-μL aliquot of supernatant was evaporated to dryness, and then derivatized with 80 μL methoxamine hydrochloride (15 mg/mL pyridine) for 90 min at 37°C with continuous shaking. Subsequently, 80 μL BSTFA with 1% TMCS and 20 μL hexane were added to the mixture and heated for 1 h at 70°C to form derivatives. After derivatization and cooling at room temperature for 30 min, the sample was used for GC-MS analysis.

### GC-MS

GC-MS was carried out on an Agilent 7890A/5975C GC/MSD system (Agilent Technologies, Santa Clara, CA, USA) and an HP-5 MS fused silica capillary column (30 m × 0.25 mm × 0.25 μm; Agilent Technologies). Each 0.5-μL aliquot of the derivatized solution was injected into the system with an injector temperature of 280°C. The MS quadrupole temperature was set at 150°C, and the ion source temperature was set at 230°C. Helium carrier gas was used at a constant flow rate of 6.0 mL/min. The column temperature was initially kept at 70°C for 2 min, and then increased from 70 to 160°C at 6°C/min. The temperature was thereafter increased from 160 to 240°C at 10°C/min, and finally increased from 240 to 300°C at 20°C/min, and maintained for 6 min. Data acquisition was performed in the full scan mode from 50 to 600 m/z. Moreover, quality control samples were prepared by mixing equal amounts of brain tissue sample and were pretreated in the same manner as the real samples. One quality control sample was inserted after every 8–10 real sample runs.

### GC-MS data analysis

The acquired MS files from GC-MS analysis were processed using TagFinder after conversion into NetCDF file format. This processing enabled deconvolution, alignment, and data reduction to produce a list of mass (m/z) and retention time (RT) pairs with corresponding intensities for all detected peaks from each data file in the data set. The resulting data set, including the peak index (RT-m/z pair), sample names (observations), and normalized peak area percentages, were imported into SIMCA-P (version 14.0; Umetrics, Umea, Sweden). Principal component analysis was used to observe the distributions of all samples and assess the stability of analysis. Orthogonal partial least-squares discriminant analysis (OPLS-DA) was conducted to differentiate the social defeat group from the control group. By analyzing OPLS-DA loadings, the differential metabolites (metabolites that differ significantly between the two groups) were identified (Variable Influence on Projection, VIP> 1 and P< 0.05)[32]. The quality of the OPLS-DA model was represented by R2X, R2Y, and Q2. R2X and R2Y were used to quantify the goodness-of-fit; Q2 was employed to assess model predictability. A 200-iteration permutation test was performed to validate the differences between the groups. The heatmap was constructed by the differential metabolites and implemented in R (version 3.2.2) with hierarchical clustering.

Metabolite annotation was performed by comparing the mass fragments with National Institute of Standards and Technology standard mass spectral databases with a similarity of > 70% and finally verified with available reference compounds. The compound annotation was also performed by comparing accurate mass and RT of reference standards in our in-house library.

### Statistical analysis

The parametric Student’s t-test and the nonparametric Mann-Whitney U-test were performed using SPSS software (version 17.0) to compare differences between the two groups in terms of the SPT, OFT, EPM and FST. A repeated measures analysis of variance (ANOVA) factoring treatment (control and social defeated) and time (baseline, week 1, 2, 3) was used to the weight gain data. The Kolmogorov-Smirnov test and Shapiro-Wilk test were used to assess the normality of the data from behavioral testing. A P-value less than 0.05 was considered to indicate statistical significance. Correlations between two parameters were assessed using Spearman's rank correlation coefficient.

### Molecular pathway and network analysis in IPA

To systematically evaluate the metabolic differences between the two groups and to identify the potential relationships among the differential metabolites, identified metabolites were subjected to IPA (Ingenuity Systems, Redwood City, CA). Accession numbers of detected metabolites (with PubChem IDs) and *P*-values from Student’s t-tests were entered into Microsoft Excel and then imported into IPA to identify the biological relationships among the metabolites. Canonical pathways and interaction networks were generated based on the knowledge sorted in the Ingenuity Pathway Knowledge base. The network score was based on the hypergeometric distribution and was calculated with the right-tailed Fisher’s exact test. The higher the score, the more relevant the eligible submitted molecules were to the network.

## Results

### Behavioral testing

**Fig 2:**
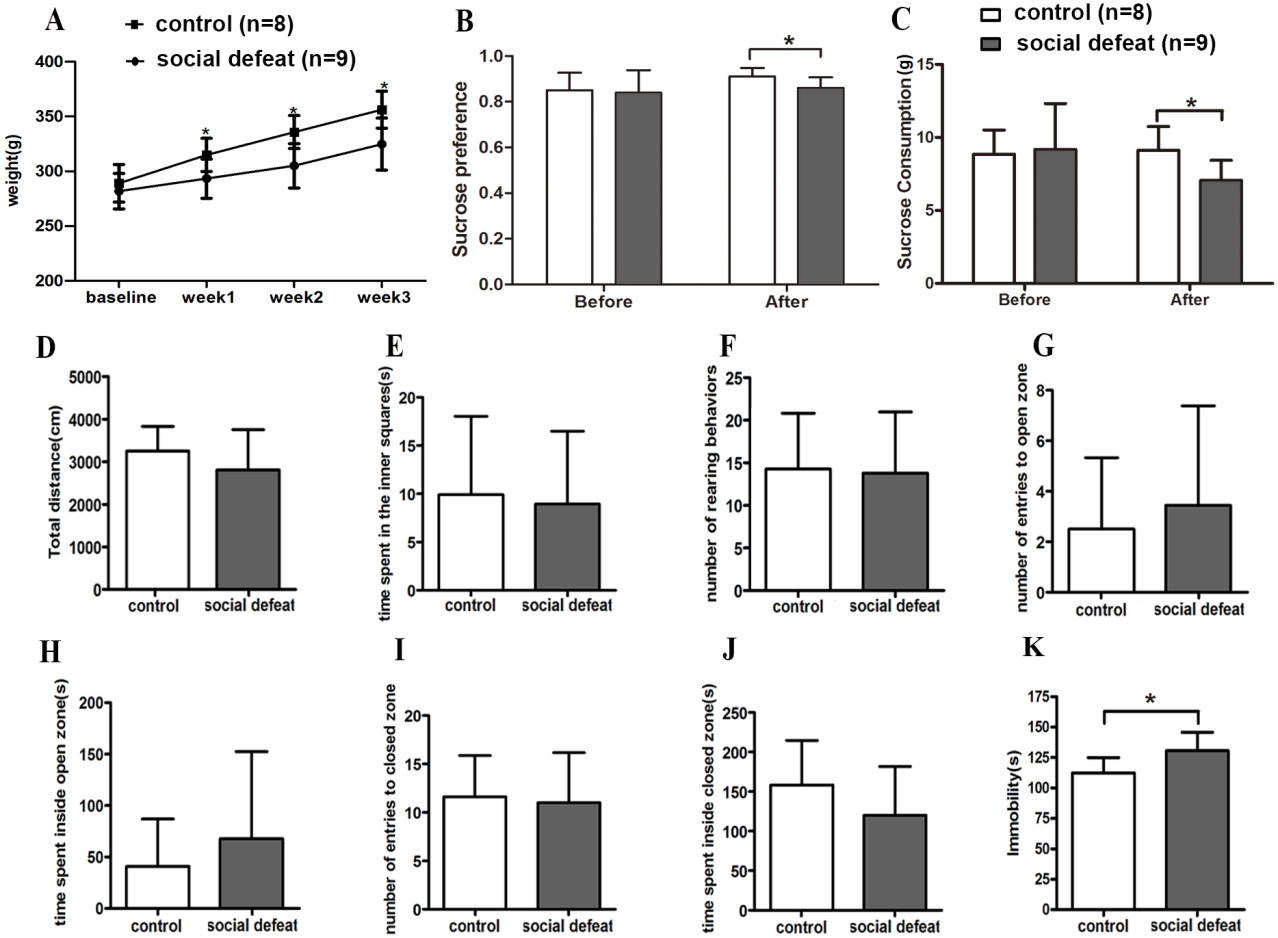
Quality assessment of the social defeat model. **(A)** Significant decreases in body weight were found inthe socially defeated group (n = 9) compared with the control group (n = 8). In the FST, the socially defeated group showed a significant decrease in sucrose preference **(B)** and sucrose consumption **(C)** compared with the control group. In the OFT, socially defeated rats did not significantly differ from control rats in total distance **(D)**, time spent in center **(E)** and number of rearing behaviors **(F)**. In the EPM, there were no significant differences between two experimental groups, including frequency **(G)** and time **(H)** spent in open arms, as well as in closed arms **(I, J)**. In the FST, longer immobility time was found in socially defeated rats **(K)**. *P< 0.05.

Seventeen SD rats were used in this study and assigned into two groups (n = 8–9 per group). Rats in the social defeat group were repeatedly exposed to the social defeat procedure for 3 weeks (Fig 1). The analysis by repeated measures ANOVA showed that the socially defeated rats gained less body weight than the control rats after 3 week (F_1.738, 26.064_ = 24.123, *P* < 0.001). After 1 weeks of stress exposure, body weight was significantly decreased in the socially defeated rats compared to control rats (F _1, 15_ = 7.131, *P* = 0.017), and this difference remained significant after 2 and 3 weeks of the social defeat procedure (F _1, 15_ = 12.398, *P* = 0.003; F _1, 15_ = 9.729, *P* = 0.007) (Fig 2A).

Anhedonia is a core symptom of depression and involves a lack of pleasure in response to rewarding stimuli. To examine the effect of social stress on depression-related behavior, we conducted the SPT before and after the social defeat procedure. At the third week, sucrose preference was significantly decreased in the socially defeated rats compared with controls (t = −2.625, *P* = 0.019; Fig 2B). Additionally, sucrose consumption decreased markedly in the socially defeated rats compared with controls (t = −2.625, *P* = 0.020; Fig 2C).

To analyze whether repeated social defeat affects other measures of depression-related behavior, we assessed the behavior of rats in the FST. In this test, the more time an animal spends immobile in a beaker of water is interpreted as a measure of depression-like behavior [33]. In the FST, immobility time was significantly elevated in socially defeated rats as compared with controls (z = −2.309, *P* = 0.021; Fig 2K), whereas no significant difference in climbing behaviors was observed between the groups (z = −1.733, *P* = 0.083).

The OFT and EPM were used to examine anxiety-like behavior in the two groups. In the OFT, total distance (t = −1.157, *P* = 0.265; Fig 2D), time spent in center (t = −0.255, *P* = 0.802; Fig 2E) and number of rearing behaviors (t = −0.141, *P* = 0.890; Fig 2F) did not differ between the socially defeated and control groups. In the EPM, there were no significant differences between the two groups regarding anxiety-related measures, including frequency (z = −0.550, *P* = 0.582; Fig 2G), and time (t = 0.800, *P* = 0.436; Fig 2H) in open arms, and frequency (t = −0.270, *P* = 0.791; Fig 2I) and time (t = −1.335, *P* = 0.202; Fig 2J) in closed arms. The results indicate that social defeat neither altered the locomotor activity nor caused anxiety-like behavior.

### Effects of social defeat on brain metabolites

**Fig 3:**
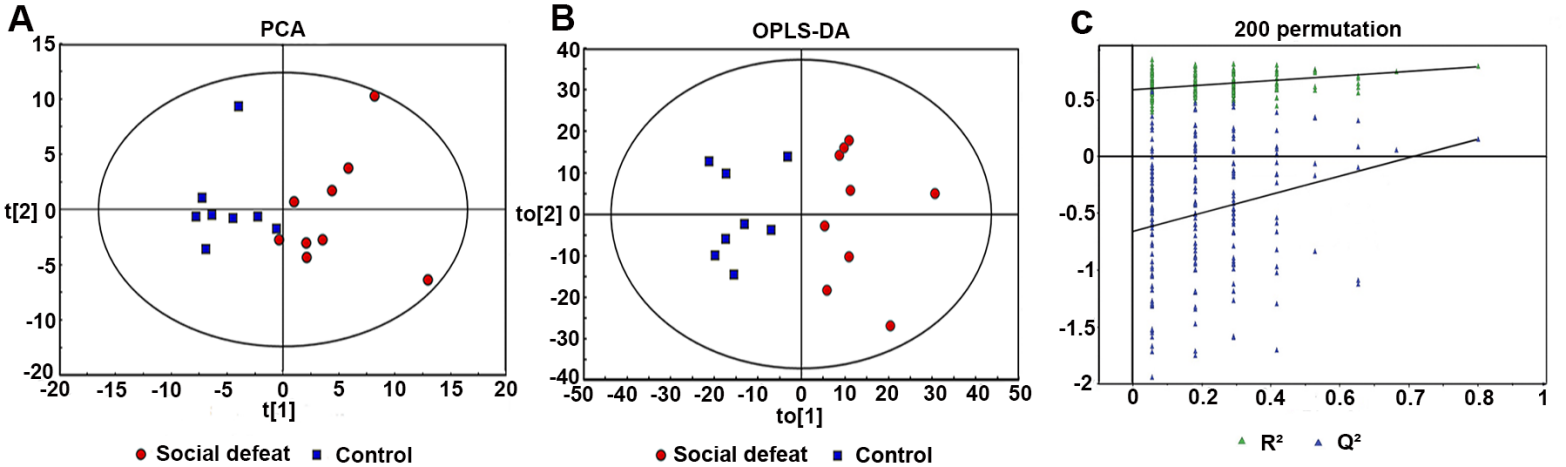
Metabolomics analysis of prefrontal cortex samples from the social defeat and control groups. **(A)**The principal components analysis score plot derived from GC-MS spectra showed clear differences between the social defeat group (n = 9) and the control group (n = 8). **(B)** Orthogonal partial least-squares discriminant analysis (OPLS-DA) model showing good predictability between the social defeat group and the control group. **(C)** The permutation plot indicated that the original OPLS-DA model was valid.

**Fig 4:**
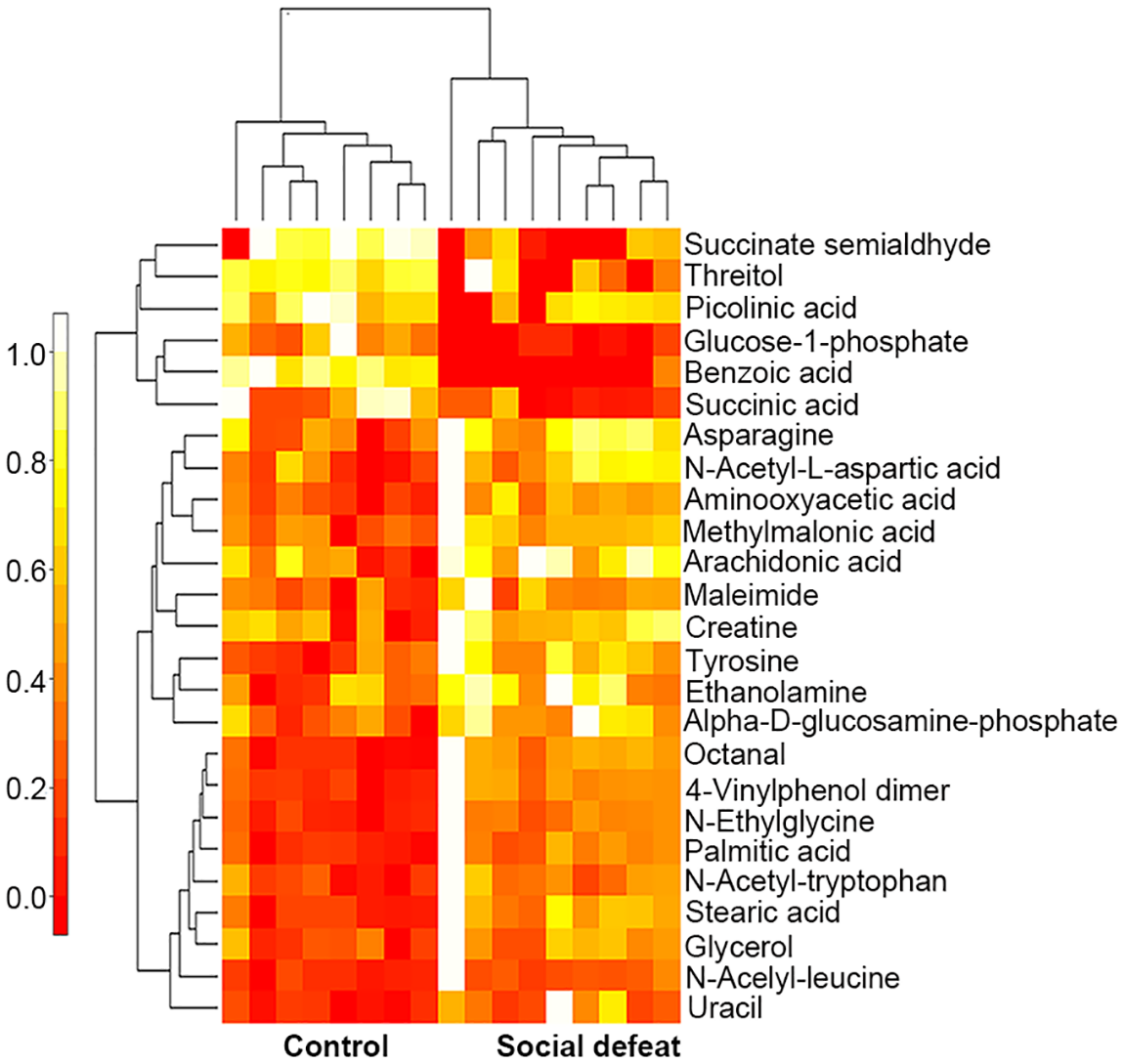
Heatmap visualization of metabolomics data for the prefrontal cortex. The heatmap was constructed based on the differential metabolites. The data of heatmap were normalized by rescaling between 0 and 1. Distinct segregation was observed between control and socially defeated rats. Rows: metabolites; columns: samples. Color key indicates metabolite expression value: white, highest; red, lowest.

Brain tissues samples from the prefrontal cortex of socially defeated rats and control rats were subjected to GC-MS metabolomics profiling. A total of 327 metabolites of known identity were detected across the two groups. The PCA score plots showed clear differences between the social defeat and control groups (R^2^X = 0.409, Q^2^ = 0.066; Fig 3A). Based on the OPLS-DA, 25 metabolites were statistically distinguishable between the two groups (R^2^X = 0.370, R^2^Y = 0.793, Q^2^ = 0.151; Fig 3B). The permutation plot indicated that the original OPLS-DA model was valid (Fig 3C). Each of the 25 significant differential metabolites met the criteria VIP > 1 and *P* < 0.05 (Table 1). Heatmap visualization of the differential metabolomics data showed distinct segregation between control and socially defeated rats (Fig 4). Five of the 25 differential metabolites are involved in lipid metabolism, five participate in amino acid metabolism, and three are involved in energy metabolism. Based on the data analysis of metabolic profiling, molecular alterations occurred in the brain of socially defeated rats.

**Table 1:** Differential metabolites in the prefrontal cortex detected by GC-MS.

No	Metabolite	VIP^a^	*p*-value^a^	FC^b^	Pathway
1	N-acetyl-L-aspartic acid	1.62	2.20×10^−3^	0.28	Amino acid metabolism
2	asparagine	1.57	2.10×10^−3^	0.15	Amino acid metabolism
3	succinate semialdehyde	1.88	3.00×10^−3^	-1.60	Amino acid metabolism
4	picolinic acid	1.13	4.03×10^−2^	-0.76	Amino acid metabolism
5	tyrosine	1.03	8.00×10^−4^	0.26	Amino acid metabolism
6	glucose-1-phosphate	2.17	1.00×10^−4^	-3.06	Energy metabolism
7	creatine	2.7	1.26×10^−2^	0.31	Energy metabolism
8	succinic acid	1.01	7.70×10^−3^	-0.89	Energy metabolism
9	octanal	1.7	1.00×10^−4^	0.48	Ethanol metabolism
10	α-D-glucosamine-phosphate	1.27	7.90×10^−3^	0.14	Carbohydrate metabolism
11	threitol	1.13	4.30×10^−3^	-1.25	Carbohydrate metabolism
12	glycerol	2.05	1.89×10^−2^	0.23	Lipid metabolism
13	stearic acid	2.17	2.00×10^−4^	0.34	Lipid metabolism
14	palmitic acid	1.76	2.20×10^−3^	0.37	Lipid metabolism
15	arachidonic acid	1.21	3.50×10^−3^	0.28	Lipid metabolism
16	ethanolamine	1.22	8.80×10^−3^	0.28	Lipid metabolism
17	methylmalonic acid	1.02	1.40×10^−3^	1.01	Nucleotide metabolism
18	uracil	1	6.10×10^−3^	0.48	Nucleotide metabolism
19	maleimide	1.02	2.60×10^−2^	0.42	Organic synthesis
20	benzoic acid	1.16	2.56×10^−9^	-4.32	Phenylalanine metabolism
21	4-vinylphenol dimer	1.22	4.00×10^−4^	0.53	Phenylpropanoid biosynthesis
22	N-acetyl-L-leucine	1.53	2.94×10^−2^	1.52	—
23	N-acetyl-tryptophan	1.12	1.39×10^−2^	0.39	—
24	N-ethyl-glycine	2.43	1.70×10^−3^	0.54	—
25	aminooxyacetic acid	2.97	1.30×10^−3^	0.55	—

^a^ Only metabolites with variable influence on projection (VIP) values greater than 1.0 and *P*-values less than 0.05 were deemed to be statistically significant.

^b^ Fold change was calculated as the average mass response (area) ratio between the two classes. The table displays the log2 transformation of the average fold changes (social defeated vs control).

Spearman’s rank correlation coefficient was calculated between differential metabolites and behavioral testing (SPT and FST). For the SPT, expression of three metabolites (glucose-1-phosphate, threitol and succinic acid) were positively correlated (*P* < 0.05), and expression of two metabolites [N-Acetyl-L-aspartic acid (NAA) and N-Ethylglycine] were negatively correlated (*P* < 0.05) with sucrose preference. For the FST, expression of four metabolites (arachidonic acid, uracil, tyrosine and maleimide) were positively correlated (*P* < 0.05), and expression of five metabolites (benzoic acid, glucose-1-phosphate, threitol, succinic acid and picolinic acid) were negatively correlated (*P* < 0.05) with immobility time. Furthermore, glucose-1-phosphate was positively correlated with sucrose preference (*P* = 0.028, r = 0.531), but negatively with the immobility time (*P* = 0.031, r = −0.524). The same trends were also observed for threitol (*P* = 0.005, r = 0.651; *P* = 0.009, r = −0.610) and succinic acid (*P* = 0.002, r = 0.704; *P* = 0.034, r = −0.517) in the SPT and FST, suggesting that changes in these metabolites are related to depression-like behavior.

### IPA analysis based on the differential metabolites

**Fig 5:**
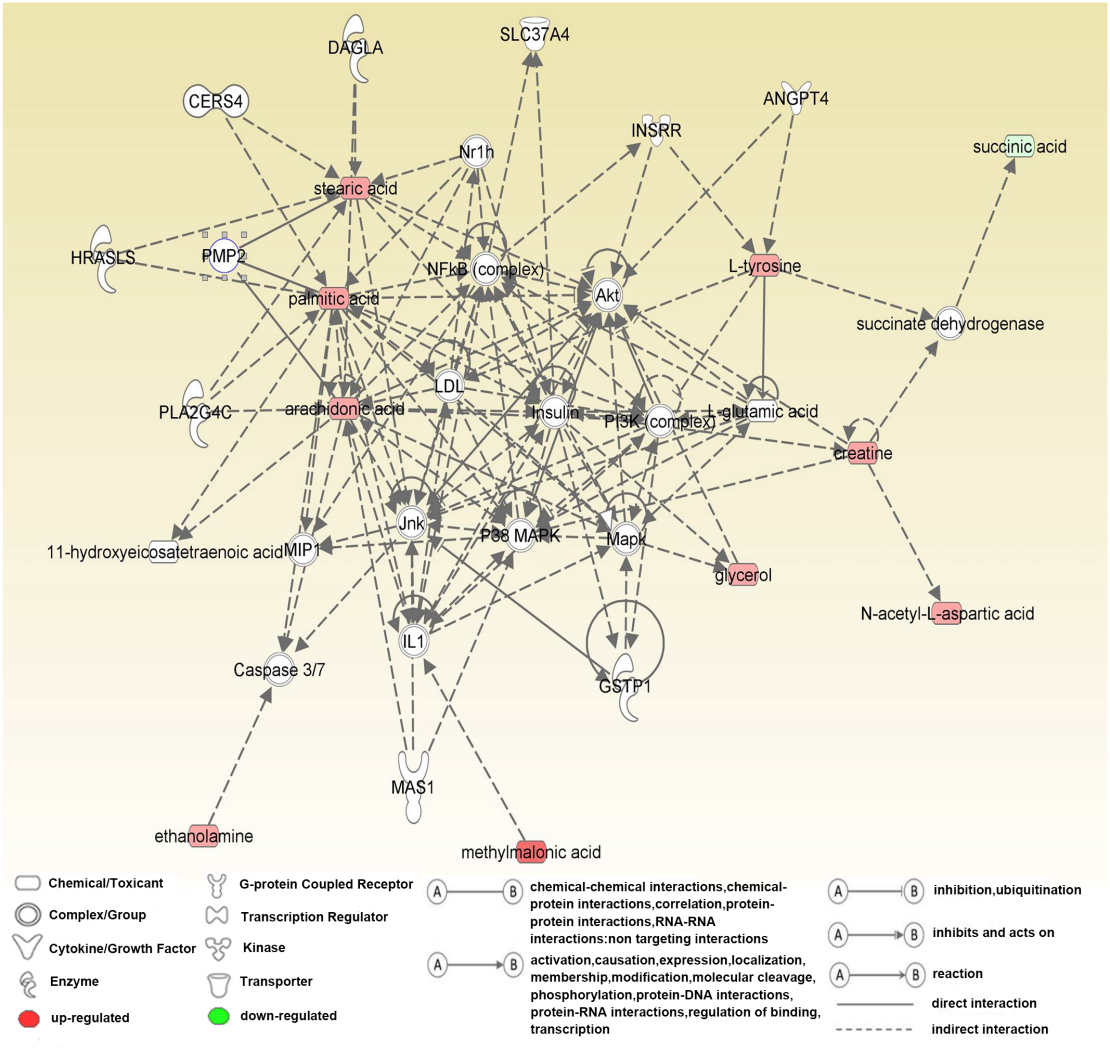
Ingenuity pathways analysis of differential metabolites. The network “Hereditary Disorder, Neurological Disease, Lipid Metabolism” was the most significantly altered network between socially defeated group (n = 9) and control group (n = 8), with a score of 26. The network includes 10 out of the 25 metabolites uploaded on the Ingenuity Pathway Analysis reference database. These metabolites are functionally related and have a role in the same network centred on NF-κB, MAPK and AKT signaling cascade. Red indicates metabolites that were upregulated, while green indicates metabolites that were downregulated, in the social defeat group compared with the control group.

To find novel relationships among the detected metabolites and to identify metabolic differences between socially defeated rats and control rats, we performed IPAs of the metabolite alterations in the prefrontal cortex. The five most significantly different canonical pathways were anandamide degradation, 4-aminobutyrate degradation I, glutamate degradation III (via 4-aminobutyrate), stearate biosynthesis I (animals), and GABA receptor signaling (Table 2). In the network function analysis, “Hereditary Disorder, Neurological Disease, Lipid Metabolism” was the most significantly altered network, with a score of 26 (Fig 5). Cellular compromise, lipid metabolism, molecular transport, small molecule biochemistry, and cell cycle were five aspects of molecular and cellular functions associated with the 25 significant metabolites.

**Table 2:** Five canonical pathways most significantly different between the socially defeated group and the control group, and the related molecular and cellular functions revealed by IPA analysis.

Canonical pathways	*p*-value^a^	Molecular and Cellular Functions	*p*-value^b^
Anandamide Degradation	5.70×10^−6^	Inflammatory Disease	2.99×10^−2^–3.46×10^−7^
4-aminobutyrate Degradation I	2.56×10^−5^	Lipid Metabolism	4.05×10^−2^–2.30×10^−7^
Glutamate Degradation III	3.75×10^−5^	Molecular Transport	4.72×10^−2^–2.30×10^−6^
Stearate Biosynthesis I (Animals)	6.84×10^−4^	Small Molecule Biochemistry	4.63×10^−2^–2.30×10^−6^
GABA Receptor Signaling	1.45×10^−3^	Cell Cycle	3.20×10^−2^–3.42×10^−6^

^a^ The p-value is calculated using the right-tailed Fisher’s exact test by IPA. A smaller the p-value represents a stronger association between the metabolites and canonical pathways of IPA.

^b^ The p-value is calculated using the right-tailed Fisher’s exact test by IPA. A smaller the p-value represents a stronger association between the molecular and cellular functions of IPA and the metabolites.

## Discussion

In this study, we induced depression-like behavior in rats by exposing them to social defeat stress. An untargeted GC-MS approach was used to identify metabolic changes in the prefrontal cortex between the socially defeated group and the control group. Half of the differential metabolites were found to be primarily involved in lipid metabolism, amino acid metabolism, and energy metabolism.

The SPT has been interpreted as a model of anhedonia-like behavior, namely a decreased interest in rewarding activities seen in patients with depression. In our study, we used both sucrose intake and sucrose preference to measure the animal’s responsiveness to a natural reward. We collected the data of the SPT at two time points. Data acquired before the induction of social stress were used to evaluate the equivalence between the two groups at baseline, and data acquired after the social defeat procedure were used to evaluate the model. Because we were primarily concerned with the results at the end point, the parametric Student’s t-test was chosen for the statistical analysis of sucrose preference [31]. The repeated measures ANOVA is more suitable for the SPT analysis as it had three time points [34]. In addition, box-plots were used to evaluate a ceiling effects, and all the animals had a preference lower than 100% (see S1 Fig). After a 3-week social defeat procedure, a clear difference emerged showing that socially defeated rats had lower sucrose preference than control rats, which suggests that repeated social defeat induced a depression-like response. However, at the end of the social defeat procedure, sucrose preference of the stressed animals was closed to their baseline levels, whereas the consumption of sucrose water was significantly less than the baseline. The small difference in sucrose preference between the two groups is presumably because of a decrease in consumption in socially defeated rats. Therefore, we also used the FST to examine depression-related behavior.

The FST has been commonly used to examine depression-like and antidepressant-like behavior in numerous pharmacological studies [33]. The small difference in immobility time between the two groups may be because of the short period of the social defeat paradigm used in this study. With a longer period, differences between the socially defeated and control rats were greater [31]. In our study, 23°C was chosen as the water temperature for the FST. However, Jefferys and Drugan et al. found that water temperatures (19°C or 20°C) lower than 25°C would result in increased immobility [35, 36]. In contrast, results of Pintér et al. have indicated that no differences were found in plasma adrenocorticotropic hormone or corticosterone concentrations following exposure of rats to the FST at 20°C, 25°C, and 30°C [37]. Thus, we considered that the effect of the differences in water temperature within this range was limited. Additionally, Drugan et al have indicated that exposured to cold water results in behavioral, physiological, and neural adaptations 24 h later, and attenuation of swim-induced c-Fos expression in serotonergic neurons [35]. In their study, the FST consisted of a 15-min swim on day 1 and a 5-min swim on day 2, and their conclusion was based on differences between day 1 and day 2. In our study, there were two important difference. First, Drugan et al used normal Wistar rats to conduct the FST, and we used the social defeat model in Sprague–Dawley rats to conduct the test. Second, Drugan et al. collected the brain tissue for immunohistochemical procedures at just two hours after the onset of the swim session on day 2, we obtained the brain tissue at day 3. Hence, we consider that the effect of the cold swim on complex changes in depression models required further study. Unlike the SPT and FST, the OFT and EPM did not show a significant difference between the two group. In contrast, Patki et al. found increased anxiety-like behavior with the OFT and EPM [38]. It is possible that the method and period of the social defeat procedure may have led to these conflicting results.

After the social defeat procedure, the untargeted GC-MS approach was used to identify metabolic changes in the prefrontal cortex. In this study, we tested the samples one by one, but did not perform repeated tests on each sample. The use of repeated independent tests on the same samples may have obtained more stable results for the GC-MS analysis.

IPA revealed that the network “Hereditary Disorder, Neurological Disease, Lipid Metabolism” was the most altered between the social defeat group and the control group. Three lipids (stearic acid, palmitic acid, and arachidonic acid), two amino acids (creatine and NAA), and two dicarboxylic acids (methylmalonic acid and succinic acid) were identified in this network. Numerous studies have reported changes in lipid metabolites in the plasma of MDD patients and in animal models of depression [25, 39, 40]. However, very few studies have examined changes in brain lipid metabolites in animal models of depression. In this GC-MS-based metabolomics study, we identified several changes in lipid metabolism in socially defeated rats compared with controls. We found significantly increased levels of palmitic acid, arachidonic acid, ethanolamine, stearic acid, and glycerol in the prefrontal cortex of socially defeated rats. Zhang et al. have reported that palmitic acid could modify biological functions including signal transduction, vesicular transport and maintenance of cellular architecture of proteins associated with neuroprogressive processes [41]. Arachidonic acid is also a key metabolite in the network, and is extensively involved in brain signaling via serotonergic, glutamatergic, dopaminergic, and cholinergic receptor stimulation, all of which are associated with major depression [42–45]. Compared with the relatively constant levels of eicosapentaenoic acid and docosahexaenoic acid in erythrocytes, plasma, and brain tissue, arachidonic acid levels appear to change more substantially in brain than in plasma or erythrocytes [46]. Modica-Napolitano et al. demonstrated that ethanolamine perturbs mitochondrial bioenergetic function by inhibiting electron transfer, suggesting a possible relationship between mitochondrial dysfunction and altered phospholipid metabolism in the brains of patients with depression [47]. Further, arachidonic acid and ethanolamine are products of the enzymatic hydrolysis of anandamide catalyzed by fatty acid amide hydrolase [48]. Previous studies have proposed that excessive breakdown of anandamide in the medial prefrontal cortex may be sufficient to produce neuropsychiatric illness, such as depression [49]. Moreover, Carnevali et al. suggested that pharmacological enhancement of anandamide signaling via inhibition of fatty acid amide hydrolase exerted antidepressive-like effects [50].

Five differential metabolites involved in amino acid metabolism (NAA, asparagine, picolinic acid, succinate semialdehyde and tyrosine) were found at higher levels in the social defeat group than in the control group. We previously found higher levels of NAA and tyrosine in the prefrontal cortex and cerebellum in a chronic unpredictable mild stress model [34, 51]. NAA is an amino acid found in all areas of the brain at high concentrations, but exclusively in neurons [52]. Baslow et al. found that NAA may play an important role in the establishment and maintenance of the nervous system [53]. Tyrosine is a precursor of dopamine, and a disturbance in tyrosine may lead to a change in dopaminergic neurotransmission in the central nervous system [54]. Indeed, depression is thought to be associated with a dysfunction of the dopaminergic reward system [55]. Asparagine is a major excitatory amino acid in the central nervous system, and increased levels of excitatory amino acids can lead to selective neuronal loss and may be involved in multiple chronic neurological disorders [56]. Picolinic acid can increase lipid peroxidation and trigger the arachidonic acid cascade, resulting in increased production of inflammatory factors [57, 58]. Moreover, picolinic acid is a metabolite of tryptophan catabolism, and tryptophan, an amino acid precursor of serotonin, plays an important role in the pathophysiology of MDD [59].

Levels of three energy metabolism-associated metabolites (creatine, succinic acid and glucose-1-phosphate) were significantly perturbed in the social defeat group compared with the control group. A similar disturbance of energy metabolism was also found in our previous studies in depression patients and chronic unpredictable mild stress model [20, 60]. Creatine is a guanidine compound synthesized from the amino acids arginine and glycine, or acquired from high-protein foods [61]. Creatine plays an important role in ATP turnover along with phosphocreatine, and it may modify depressive behavior by buffering metabolic processes to prevent energy exhaustion and neuronal death [62]. In this study, creatine levels were relatively high in the social defeat group, whereas succinic acid levels were relatively high in the control group. Succinic acid is a key metabolite in the tricarboxylic acid cycle, and lower levels may suggest reduced ATP biosynthesis. Cao et al. identified ATP as an important factor in the astrocytic modulation of depressive-like behavior in adult mice, especially in the brains of chronic socially defeated mice [63].

## Conclusions

In summary, this study suggests that depression-like behavior can be caused by repeated social defeat in rats. In this process, stress-induced alterations of low molecular weight metabolites were observed in the prefrontal cortex of rats. In particular, lipid metabolism, amino acid metabolism, and energy metabolism were significantly perturbed.

## Supporting information

S1 Fig Box-plots of the SPT. (A) The box-plots of sucrose preference on the baseline. (B) The box-plots of sucrose preference after the social defeat procedure for 3 weeks.
(TIF)
